# Supplementary material for: Indole Pulse Signalling Regulates the Cytoplasmic pH of E. coli in a Memory-Like Manner
Source: Sci Rep. 2019 Mar 7;9:3868. doi: 10.1038/s41598-019-40560-3 (PMC6405993; doi:10.1038/s41598-019-40560-3)
Supplement: Supplementary file 1 — Supplementary Information [file 41598_2019_40560_MOESM1_ESM.pdf]

SUPPLEMENTARY INFORMATION

Indole Pulse Signalling Regulates the Cytoplasmic pH of *E. coli* in a Memory-Like Manner

Ashraf Zarkan[1], Santiago Caño Muñiz[1,2], Jinbo Zhu[3], Kareem Al Nahas[3], Jehangir Cama[3], Ulrich F. Keyser[3], and David K. Summers[1,*]

[1]Department of Genetics, University of Cambridge, Cambridge, CB2 3EH, UK

[2]MRC Laboratory of Molecular Biology, Cambridge, CB2 0QH, UK

[3]The Cavendish Laboratory, Department of Physics, University of Cambridge, Cambridge, CB3 0HE, UK

*Correspondence: dks11@cam.ac.uk

Supplementary Methods

Concentrating indole with C18 columns

Indole production in *E. coli* occurs mostly during the transition from exponential to stationary phase[1]. To measure the low concentration of indole accurately during lag and exponential phase, a pre-concentration step was added before the Kovacs assay, using C18 solid phase extraction (SPE) cartridges (SampliQ C18, Agilent, CA, USA). C18 SPE cartridges concentrate low quantities of non-polar analytes, and their use for concentrating various indole analogues has already been established[2].
The procedure was performed according to the manufacturer's instructions with minor modifications. Before use, each cartridge (500 mg octadecylsilane – 6 ml) was equilibrated by flowing-through 10 ml 1-pentanol, followed by 10 ml deionised water. A sample (50 ml) from a lag-phase or exponential-phase bacterial culture was taken, and cells harvested by centrifugation at 2755 x g for 10 min (Eppendorf 5810 R centrifuge). The supernatant was flowed through an equilibrated cartridge and the cartidge was then washed with 10 ml deionised water. Indole was eluted from the cartridge with 5 ml 1-pentanol, resulting in an eluate containing indole concentrated ten-fold.

Kovacs assay

The Kovacs assay is an established technique providing consistent results from different laboratories[3,4]. The assay was performed as previously described[1] to determine the presence of indole in culture supernatants. Briefly, a sample (1 ml) from a stationary-phase culture was removed, and cells harvested by centrifugation at 11337 x g for 3 min (Eppendorf Minispin microfuge). The supernatant was removed and assayed: 300 µl of Kovacs reagent (10 g of *p*-dimethylamino-benzaldehyde dissolved in a mixture of 50 ml of HCl and 150 ml of amyl alcohol) was added to the supernatant and incubated for 2 minutes. The presence of indole was indicated by the formation of red colour following the addition of

Kovacs reagent.

The Kovacs assay was also used to determine the concentration of indole in lag and exponential phase culture supernatants, after a pre-concentraion step with C18 columns. The assay was performed in a 96-well plate as previously described[5]. Briefly, 100 µl of x10 indole concentrated samples (in 1-pentanol) were incubated with 150 µl of Kovacs reagent for 5 min at room temperature. The reaction produced a soluble red product, which was measured spectrophotometrically at 530 nm (SpectraMax 190 Microplate reader; Molecular Devices, CA, USA). At least six known indole concentrations from 0 to 300 µM (in 1-pentanol) were assayed in triplicate and the mean results were used to construct a standard curve. Indole concentrations in unknown samples (also tested in triplicate) were calculated by comparison.

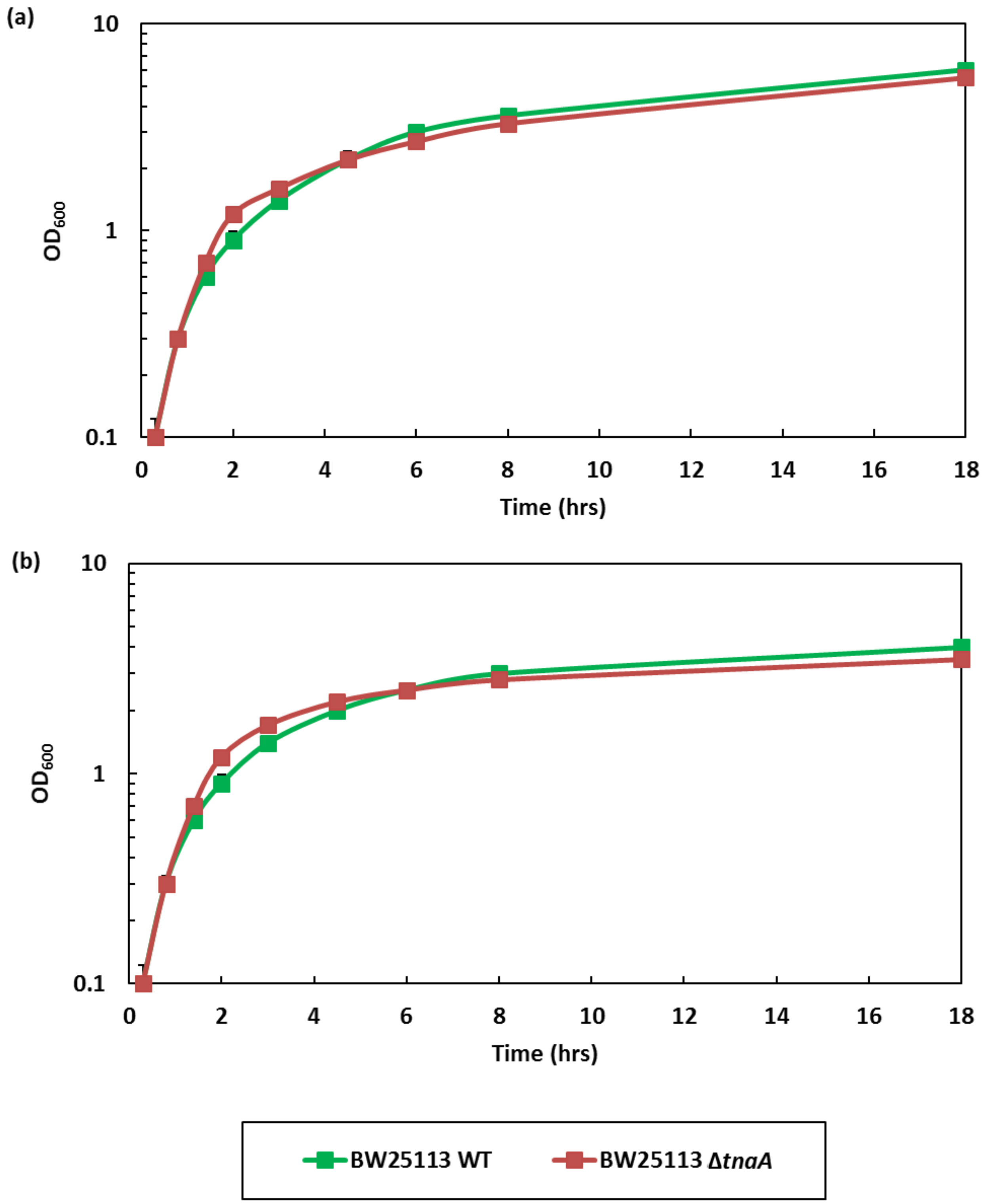

Supplementary Fig. S1: Growth curves of *E. coli* BW25113 WT and Δ*tnaA* growing in (a) LB and (b) M9/Glucose (0.4%). Overnight cultures were diluted to OD_{600} = 0.05 in fresh LB or M9 and cultures were grown at 37 °C and sampled at intervals between OD_{600} = 0.1 – 0.6 for LB and OD_{600} = 0.1 – 0.4 for M9. The final sample was taken after overnight incubation (~ 18 hrs). The transition between exponential to stationary phase was observed around the same time (~ 2 hrs after sub-culture) in both media.

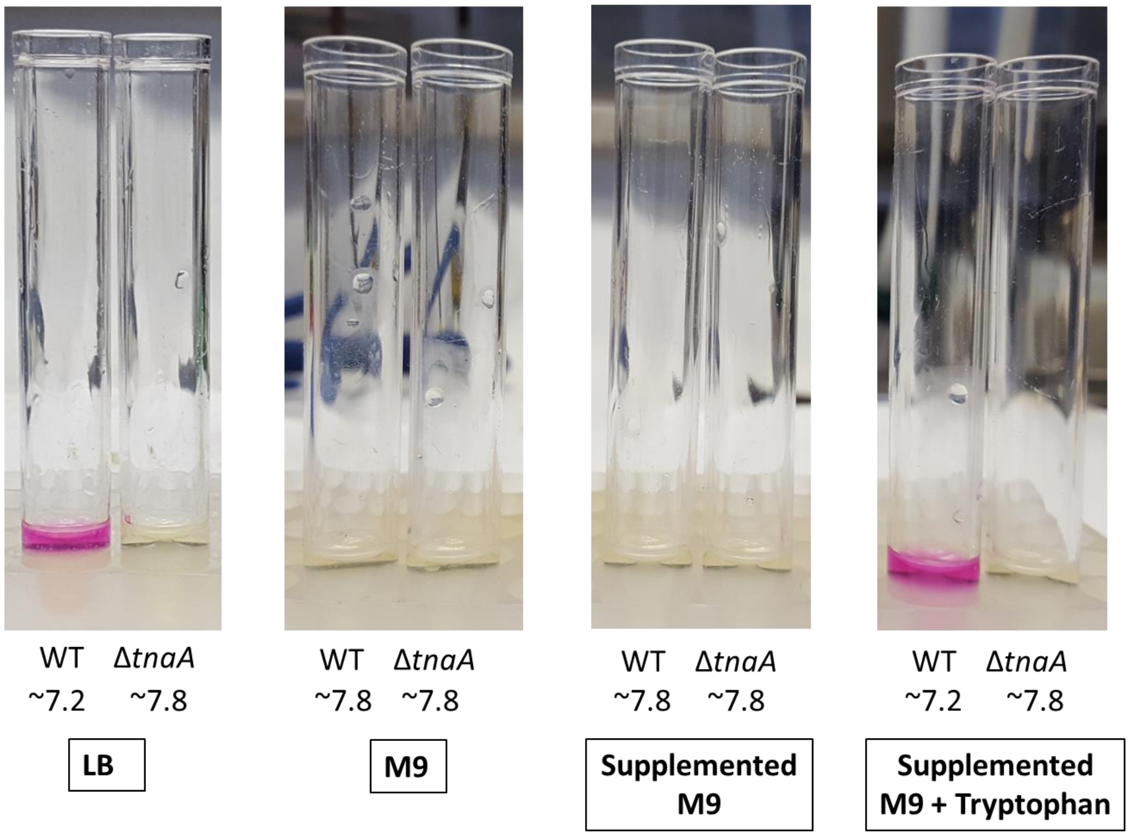

Supplementary Fig. S2: The absence of indole production correlates with the higher cytoplasmic pH. The cytoplasmic pH of the WT *E. coli* is ~ 7.2 when indole is produced and ~ 7.8 when indole is absent. The mutant strain lacks the ability to convert tryptophan to indole under any conditions so the cytoplasmic pH is always ~ 7.8. The presence of indole is indicated by the formation of red colour following the addition of Kovacs reagent. Samples were taken from stationary-phase cultures of BW25113 WT and BW25113 Δ*tnaA* grown in four different media: LB, M9 (containing glucose as a carbon source), supplemented M9 (glucose, Vitamin B1, trace elements & casamino acids) and supplemented M9 + tryptophan. The average cytoplasmic pH (Fig. 1) is added for comparison.

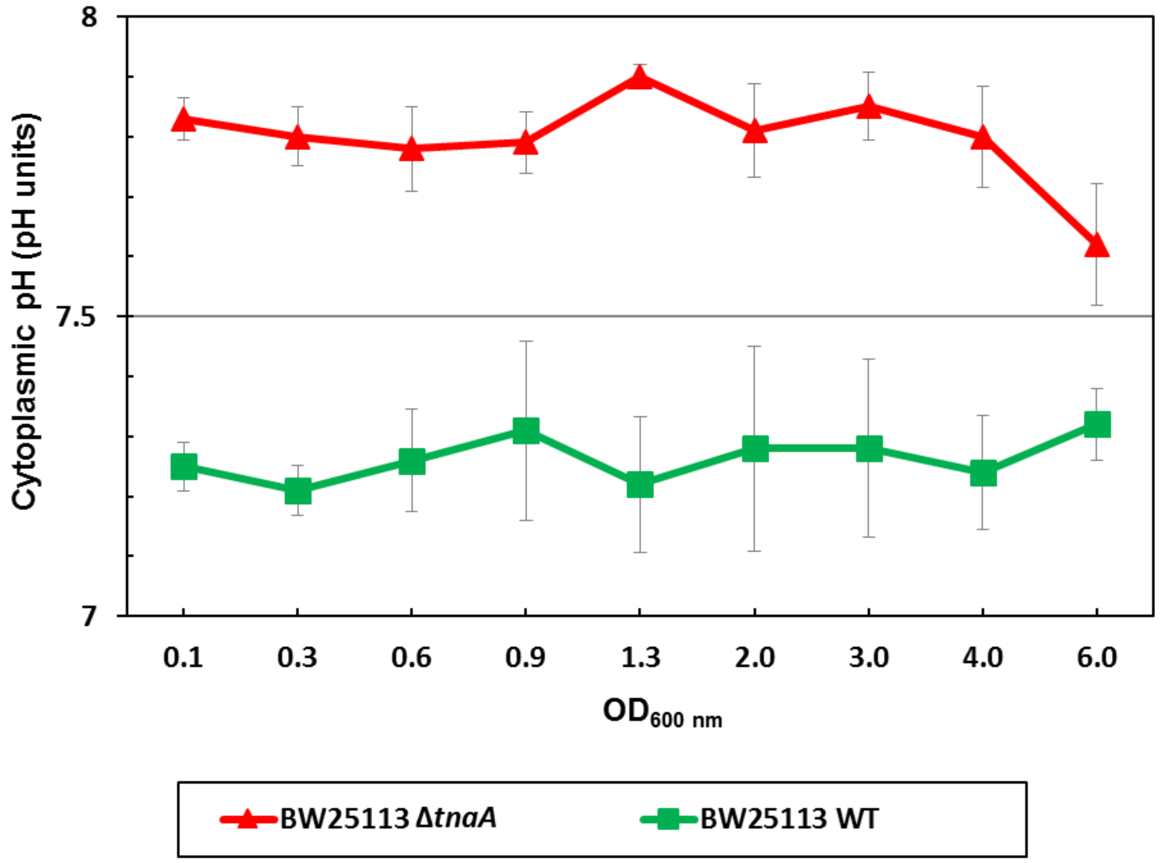

Supplementary Fig. S3: The cytoplasmic pH of *E. coli* BW25113 WT and Δ*tnaA* growing in LB, measured by flow cytometry . Cultures of BW25113 and BW25113 Δ*tnaA* in LB were sampled throughout exponential and stationary phase, and samples were analysed by flow cytometry. The cytoplasmic pH of each sample was determined by the average fluorescence intensity of pHluorin normalised to mCherry and compared to a standard curve. pH values are in agreement with those obtained by fluorescence spectroscopy (Fig. 1). Data are presented as means ± SD, and the final point in each line is an overnight sample (~ 18 hrs incubation).

.

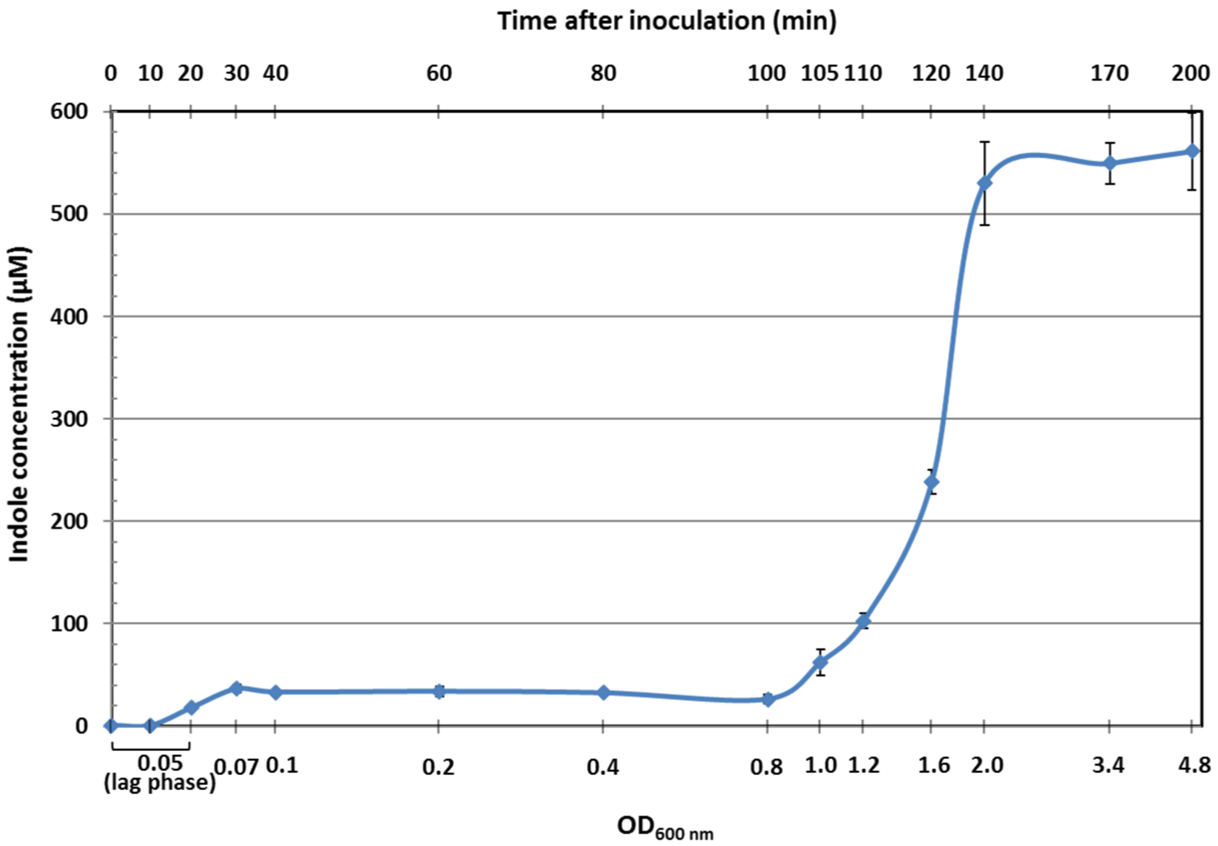

Supplementary Fig. S4: Indole concentration in the LB supernatant of *E. coli* BW25113 WT during lag, exponential and stationary phase. An overnight culture of BW25113 WT in LB was subcultured into fresh LB (OD_{600} = 0.05). Samples were taken throughout the lag phase, which lasted ~ 20 min, the exponential phase and the stationary phase. For samples from lag- and exponential phase, Indole was measured by Kovacs assay after passing each sample through a C18 solid phase extraction column, which concentrated indole 10-fold. For samples from stationary phase (OD600 > 1), indole was measured by Kovacs assay without the pre-concentration step. 30 µM (± 10 µM) indole was made during the lag phase and this concentration remained constant throughout exponential phase. The first sample during the lag phase was taken immediately after sub-culturing (time after inoculation = 0 min) and had no detectable indole. Consistent with previous reports, (see, Figure 1 in Supplementary Reference 6) an increase in the supernatant indole concentration of 550 µM (± 50 µM) was seen during the transition from exponential to stationary phase, and this concentration remained constant throughout stationary phase.

Supplementary Table 1: Primers used to generate the pSCM001 plasmid using Gibson Assembly

Primer Name	Sequence (5'-3')
pBAD TOPO_FWD	ACATGGCATGGATGAACTATACAAATGAAAACGGTCTCCAGCTTGGCT
pBAD TOPO_REV	TCGCCATGTTGTCTTCTTCGCCTTTTGAAACCATGGTATGTATATCTCCTTCTTAAAGTTAAACA
mCherry_FWD	AAGAAGGAGATATACATACCATGGTTTCAAAAGGCGAAGAAGACAACATGGCGATTATCAAGG
mCherry_REV	ATagagccgccagagccgccGTACAGTTCATCCATACCGCCGGTAGAGT
pHluorin_FWD	ACggcggctctggcggctctATGAGTAAAGGAGAAGAACTTTTCA
pHluorin_REV	ACAGCCAAGCTGGAGACCGTTTTCATTTGTATAGTTCATCCATGCCA

.

Small caps: (Gly-Gly-Ser) x2 Bridge

Supplementary References

1. Gaimster, H. & Summers, D.K. Regulation *of Indole Signalling during the Transition of E. coli from Exponential to Stationary Phase. PLoS One*. **10**, e0136691 (2015).
2. Yong, J.W.H., Ge, L., Wong, W.S., Ma, Z. & Tan, S.N. Analyses of Indole Compounds in Sugar Cane (*Saccharum officinarum* L.) Juice by High Performance Liquid Chromatography and Liquid Chromatography-Mass Spectrometry after Solid-Phase Extraction. *Separations* **4**, doi:10.3390/separations4010007 (2017).
3. Chant, E.L. & Summers, D.K. Indole signalling contributes to the stable maintenance of *Escherichia coli* multicopy plasmids. *Mol. Microbiol* **63**, 35–43 (2007).
4. Li, G. & Young, K.D. Indole production by the tryptophanase TnaA in *Escherichia coli* is determined by the amount of exogenous tryptophan. *Microbiol* **159,** 402–410 (2013).
5. Darkoh, C., Chappell, C., Gonzales, C. & Okhuysen, P. A Rapid and Specific Method for the Detection of Indole in Complex Biological Samples. *Appl. Environ. Microbiol* **81**, 8093-8097 (2015).
6. Gaimster, H., Cama, J., Hernández-Ainsa, S., Keyser, U.F. & Summers, D.K. The Indole Pulse: A New Perspective on Indole Signalling in *Escherichia coli*. *PLoS One*. **9**, e93168 (2014).
